# Skeletal muscle quantity and quality evaluation in heart failure: comparing thoracic versus abdominopelvic CT approaches

**DOI:** 10.1007/s10554-024-03169-w

**Published:** 2024-07-04

**Authors:** Saeid Mirzai, Ian Persits, Pieter Martens, Jerry D. Estep, W. H. Wilson Tang, Po-Hao Chen

**Affiliations:** 1 Department of Internal Medicine, Cleveland Clinic, Cleveland, OH USA; 2 Department of Internal Medicine, Section on Cardiovascular Medicine, Wake Forest University School of Medicine, Winston-Salem, NC USA; 3 Kaufman Center for Heart Failure Treatment and Recovery, Heart Vascular and Thoracic Institute, Cleveland Clinic, Cleveland, OH USA; 4 Department of Cardiology, Cleveland Clinic Florida, Weston, FL USA; 5 Section of Musculoskeletal Imaging, Diagnostics Institute, Cleveland Clinic, Cleveland, OH USA; 6 Section of Musculoskeletal Imaging Diagnostics Institute, Lerner College of Medicine at Case Western Reserve University, Cleveland Clinic, 9500 Euclid Avenue, Desk JJ36, Cleveland, OH 44195 USA

**Keywords:** Sarcopenia, Myopenia, Heart failure, Computed tomography, Intermuscular adipose tissue

## Abstract

Given the critical role of skeletal muscle in healthy aging, low muscle mass (myopenia) and quality (myosteatosis) can be used as predictors of poor functional and cardiometabolic outcomes. Myopenia is also a part of sarcopenia and malnutrition diagnostic criteria. However, there is limited evidence for using chest computed tomography (CT) to evaluate muscle health. We aimed to compare chest CT landmarks to the widely used L3 vertebra for single-slice skeletal muscle evaluation in patients with heart failure (HF). Patients admitted for acute decompensated HF between January 2017 and December 2018 were retrospectively analyzed. Body composition measurements were made on CT of the chest and abdomen/pelvis with or without contrast one month before discharge. Skeletal muscle index (SMI) and intermuscular adipose tissue percentage (IMAT%) were calculated at several thoracic levels (above the aortic arch, T8, and T12) and correlated to the widely used L3 level. A total of 200 patients were included, 89 (44.5%) female. The strongest correlation of thoracic SMI (for muscle quantity) and IMAT% (for muscle quality) with L3 was at the T12 level (*r* = 0.834, *p* < 0.001 and *r* = 0.757, *p* < 0.001, respectively). Cutoffs to identify low muscle mass for T12 SMI (derived from the lowest sex-stratified L3 SMI tertile) were 31.1 cm²/m² in men and 26.3 cm²/m² in women. SMI and IMAT% at T12 had excellent correlations with the widely used L3 level for muscle quantity and quality evaluation in patients with HF.

**Supplementary Information:**

The online version contains supplementary material available at 10.1007/s10554-024-03169-w.

## Introduction

Skeletal muscles play a critical role in healthy aging. Beyond enabling daily activity and exercise, they hold several essential metabolic functions, including serving as the principal tissue for insulin-stimulated glucose disposal, anti-inflammatory myokine production, protein regulation, and maintenance of resting energy expenditure [1, 2]. Consequently, the loss of skeletal muscle mass, commonly termed myopenia, can have significant functional and metabolic consequences [3]. These include exacerbated cardiometabolic disease, exercise intolerance, disability, diminished quality of life, hospitalizations, and even death [4].

Given skeletal muscles’ diverse functions, myopenia is a critical component of the diagnostic criteria for sarcopenia and malnutrition [5, 6]. Like sarcopenia, which is defined as the loss of skeletal muscle mass (myopenia) and strength (dynapenia), myopenia can be primary (age-related) or secondary to factors other than aging, including chronic diseases such as heart failure (HF) [3]. Current literature reveals a significant discrepancy between myopenia and dynapenia, mainly attributable to reliance on dual-energy X-ray absorptiometry (DXA) and bioelectrical impedance analysis (BIA) as proxies for lean mass without directly evaluating muscle quantity or quality [7, 8].

Single-slice skeletal muscle area (SMA) has been shown to correlate with total body muscle mass and muscle strength [8, 9]. It also predicts poor outcomes in various clinical settings, with computed tomography (CT) and magnetic resonance imaging (MRI) considered gold-standard methods [10]. With much of the knowledge gained in oncology and hepatology, the current best practice is total muscle or psoas-only muscle quantification at the third or fourth lumbar vertebral levels [11]. However, this practice is of limited utility in heart or lung diseases, where imaging is less frequently obtained in the abdomen/pelvis or lumbar spine relative to the chest. Unfortunately, studies comparing thoracic-level to lumbar-level muscle measurements have been rare in cardiology and non-existent in HF.

Identifying an optimal thoracic landmark for skeletal muscle assessment could make possible opportunistic screening in patients with HF, who both tend towards functional decline and associated morbidity and commonly undergo thoracic CT evaluation. To that end, we sought to evaluate correlations between skeletal muscle quantity and quality measurements at various thoracic landmarks compared to the widely-used muscle landmarks at the L3 level. We aimed to determine the best surrogate with sex-stratified cutoff values in patients with HF.

## Materials and methods

The Cleveland Clinic Institutional Review Board approved this study. Given the retrospective design, the need for written informed consent was waived.

**Fig. 1:**
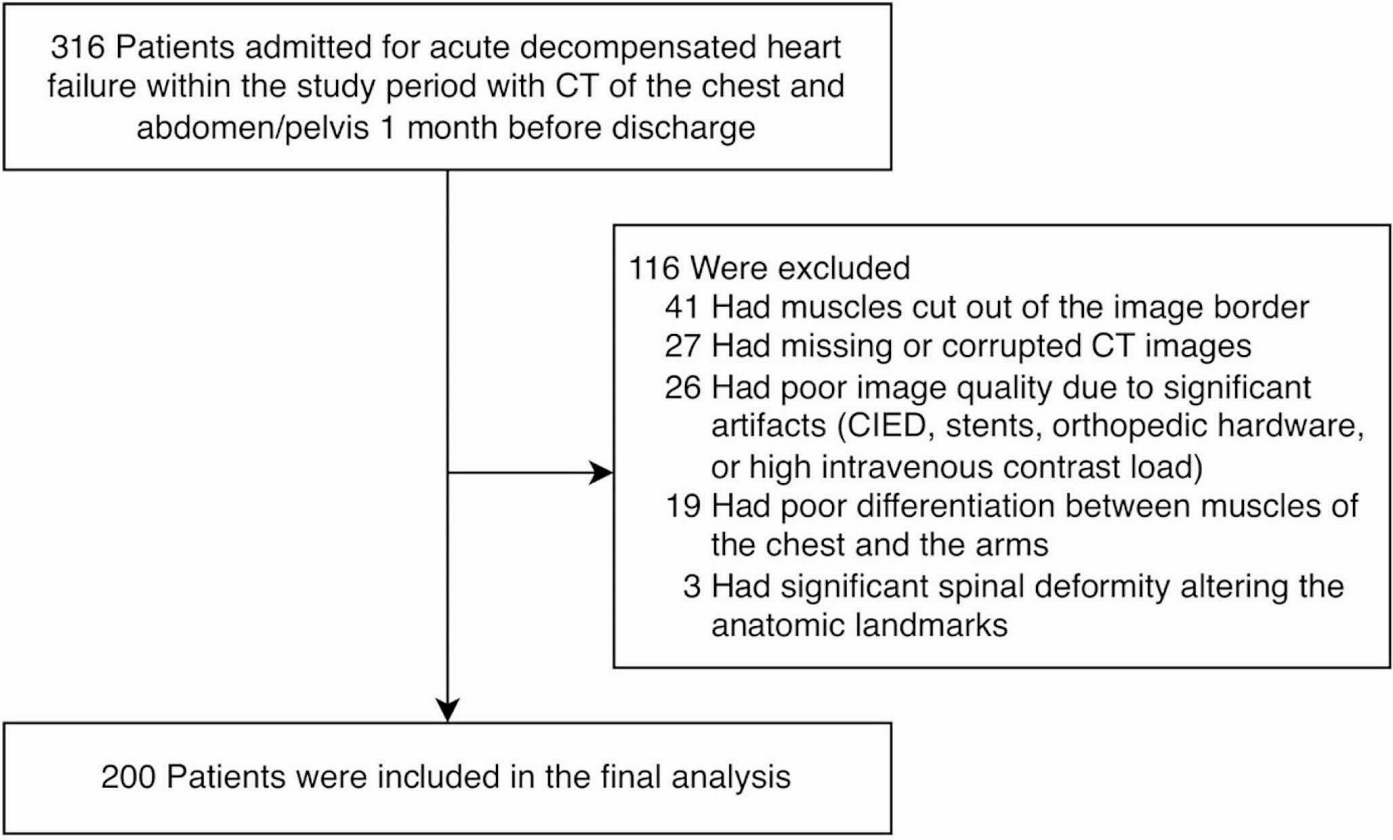
Patient selection. CT, computed tomography; CIED, cardiac implantable electronic device

Patients admitted to the Cleveland Clinic between January 2017 and December 2018 for a primary diagnosis of acute decompensated HF (ADHF) were retrospectively identified. ADHF was defined as an admission lasting > 24 h with signs and symptoms of congestion requiring intravenous diuretics. Patients with a history of HF or de novo HF were eligible for the current study irrespective of their admission left ventricular ejection fraction (EF). The main inclusion criterion was the presence of both CT imaging of the chest and abdomen/pelvis with or without contrast one month before the discharge date. Both contrast and non-contrast CTs were eligible as prior research has shown that SMA is minimally affected by contrast enhancement [12]. Exclusion criteria were primarily driven by issues with image extraction, quality, or muscles being cut off the image border or poorly differentiated from arm muscles (Fig. 1). Baseline features, including patient characteristics, comorbidities, medications, and laboratory tests, were collected retrospectively, as previously reported [13]. HF was classified based on the most current transthoracic echocardiogram into reduced (≤ 40%), mildly reduced (41–49%), and preserved (≥ 50%) EF; previous measurements were not collected for identification of improved EF.

Body composition measurements were made on opportunistic CT axial images using the commercially available software Slice-O-Matic (Version 5.0, Tomovision, Quebec, Canada) and Automatic Body composition Analyzer using Computed tomography image Segmentation plus (ABACS+) module (Voronoi Health Analytics, Vancouver, British Columbia). ABACS + is a semi-automated segmentation tool that tags skeletal muscles using its knowledge of muscle shapes at the specific level and previously validated Hounsfield unit (HU) range of -29 to 150; this is followed by tagging the intermuscular, subcutaneous, and visceral adipose tissues using corresponding predefined HU ranges [14].

Two observers (S.M. and I.P.) made measurements within the institution network blinded to patient history and outcomes. Before initiation, both observers were instructed by a board-certified radiologist (P.H.C.) on identifying anatomic landmarks and regions of interest. Approximately five hours of training with Slice-O-Matic and ABACS + was done remotely with software developers. The observers each made measurements on 100 patients, with confirmation of a board-certified radiologist on accuracy (initial 20 measurements). Intra-observer variability was assessed using the intraclass correlation coefficient (ICC) on ten randomly selected patients, equating to 50 individual measurements. On a scale of 0 to 1, ICC scores were generated to assess observer agreement; a score greater than 0.90 was considered excellent reliability, 0.75 to 0.9 good, 0.5 to 0.75 moderate, and less than 0.5 poor. Intra-observer agreements for SMA and intermuscular adipose tissue area (IMAT) were excellent at all levels besides pectoralis unilateral AbvAoAr IMAT, which was moderate and nonsignificant (Table S1). Interobserver variability was not performed given the semi-automatic nature of the measurements with ABACS+.

**Fig. 2:**
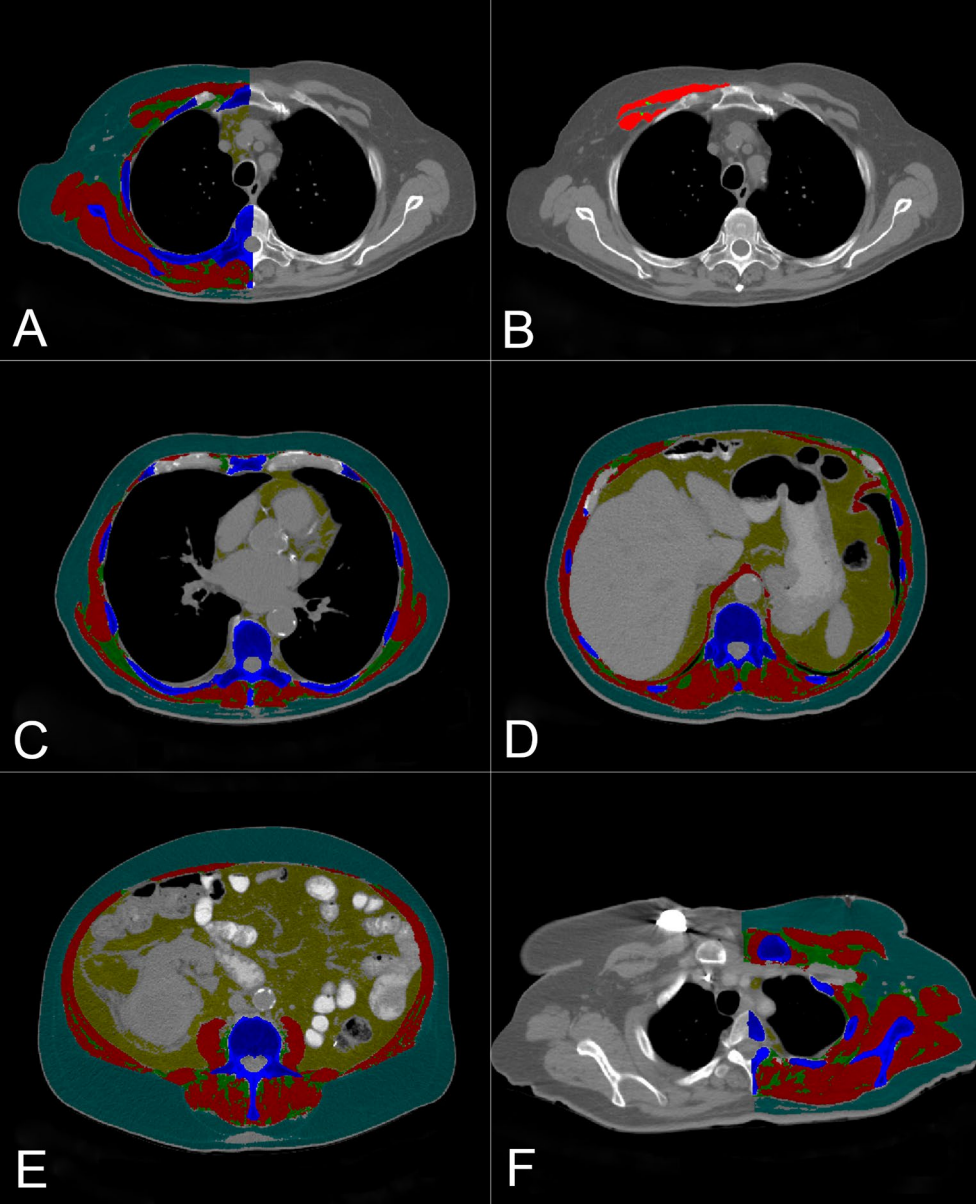
Tagged computed tomography axial images above the aortic arch (AbvAoAr; (**A**), unilateral total; (**B**), unilateral pectoralis) and at the eighth thoracic (T8; **C**), twelfth thoracic (T12; **D**), and third lumbar (L3; **E**) vertebrae. Measurements AbvAoAr were made unilaterally on the right unless a right-sided device was present (**F**). The tagged tissues were skeletal muscle (red), intermuscular adipose tissue (green), subcutaneous adipose tissue (teal), and visceral adipose tissue (yellow)

For segmentation, the CT image files were extracted and uploaded into Slice-O-Matic, where sagittal views were reproduced from axial slices. The thoracic vertebrae were manually identified by locating the most cranial vertebra with protruding ribs attached anteriorly to the sternum, identified as the first thoracic vertebra, and counting down. The lumbar vertebrae were manually identified by locating the sacrum and counting up to the last thoracic vertebra with protruding ribs, with the highest lumbar-like vertebra considered the first lumbar vertebra. Following the identification of vertebral levels on sagittal view, automated measurements of total SMA and IMAT were made on axial view immediately above the aortic arch (AbvAoAr; Fig. 2a) and the mid-vertebral body of the eighth thoracic vertebra (T8; Fig. 2c), T12 (Fig. 2d), and L3 (Fig. 2e). In addition, manual SMA and IMAT measurements were made of the pectoralis major and minor unilaterally at AbvAoAr using the ranges of -29 to + 150 HU and − 190 to -30 HU, respectively (Fig. 2b).

Measurements AbvAoAr (both automatic and manual) were unilateral right-sided, given the prevalence of cardiovascular implantable electronic devices (CIEDs) in patients with HF on the left chest, but the left side was used when issues were encountered on the right side (Fig. 2f). Frequent reasons for left-sided measurements included right chest devices and hardware, poor chest and arm muscle differentiation, muscle of interest being cut out of the right image border, and high right intravenous contract load. Measurements at all other vertebral levels were bilateral. A subgroup analysis was performed comparing the correlation of total unilateral AbvAoAr to L3 measurements in patients with and without CIEDs to assess the effect of device presence.

Following the measurement of raw values, skeletal muscle index (SMI; cm^2^/m^2^) was calculated to normalize for body size by dividing the SMA (cm^2^) by the square of the patient’s height (m^2^) [15, 16]. Two definitions of low muscle mass were used: (1) the lowest sex-stratified L3 SMI tertile, and (2) L3 SMI cutoff values of ≤ 52.4 cm^2^/m^2^ in men and ≤ 38.5 cm^2^/m^2^ in women based on frequently used cancer cutoffs [17]. The degree of myosteatosis (skeletal muscle fat infiltration) was assessed via quantification of adipose tissue within muscle fascia, also known as IMAT, which was then used to calculate IMAT% with the formula: IMAT (cm^2^) / (SMA (cm^2^) + IMAT (cm^2^)) × 100 [18].

For statistical analysis, continuous variables were expressed as mean ± standard deviation if normally distributed or median (25-75th percentile) if non-normally distributed. Categorical values were reported as numbers and percentages. The student *t-test* and analysis of variance were used for the comparison of continuous normally distributed variables, and the Kruskal-Wallis and Mann-Whitney U tests for non-normally distributed variables. The Chi-squared test was used for categorical variables. Distributional histograms were used to assess the normality of distribution. The relationships between the different vertebral level body composition measurements were assessed using the Pearson correlation coefficient (r). Fisher Z-transformation was used to determine differences in correlation strength. Cutoffs for low muscle mass were obtained using receiver operating characteristic (ROC) curve analysis to maximize true-positive and minimize false-negative diagnoses from the lowest sex-stratified tertile and the frequently used cancer L3 SMI cutoffs. A P-value equal to or less than 0.05 indicated a statistically significant difference. All statistical analyses were performed using SPSS (Version 25, SPSS Inc., Chicago, IL, USA).

## Results

Of the 316 patients who met inclusion criteria, 116 were excluded (Fig. 1). A total of 200 patients were included, 89 (44.5%) female. Overall, the characteristics of the population are reflected in Table 1. The average age was 71 ± 14, with most patients being Caucasian (75.5%). The majority had HF with preserved EF of ≥ 50% (49.0%), followed by reduced ≤ 40% (39.5%) and mildly reduced 41–49% (11.5%).

**Table 1:** Baseline characteristics of the total analytic cohort

Parameters	Total cohort (*n* = 200)
**Characteristics**	
Age (years)	71 ± 14
Female sex	89 (44.5%)
Race	
Caucasian	151 (75.5%)
Black	40 (20.0%)
Other	9 (4.5%)
Height (cm)	169.2 ± 10.9
Weight (kg)	81.5 ± 21.5
Body surface area (m^2^)	1.94 ± 0.28
Body mass index (kg/m^2^)	28.5 ± 7.1
LV ejection fraction	
Preserved	98 (49.0%)
Mildly reduced	23 (11.5%)
Reduced	79 (39.5%)
NT-proBNP (pg/mL)	5016 (1849–10,356)
Hemoglobin A1c (%)	6.4 ± 1.3
Low-density lipoprotein (mg/dL)	74.2 ± 34.1
Albumin (g/dL)	3.4 ± 0.6
**Comorbidities**	
Hypertension	173 (86.5%)
Hyperlipidemia	147 (73.5%)
Diabetes	100 (50.0%)
Coronary artery bypass grafting or percutaneous coronary intervention	43 (21.5%)
Peripheral arterial disease	66 (33.0%)
Chronic kidney disease	85 (42.7%)
Atrial fibrillation	95 (47.5%)
Cardiac resynchronization therapy	7 (3.5%)
Chronic obstructive pulmonary disease	83 (41.5%)
Cirrhosis	16 (8.0%)
Cancer history	81 (40.5%)
Smoking	
Active	39 (20.5%)
Prior	92 (48.4%)
Never	59 (31.1%)
**Medications**	
Statin	108 (54.0%)
Angiotensin-converting enzyme inhibitors or angiotensin receptor blockers	86 (43.0%)
Beta-blocker	118 (59.0%)
Mineralocorticoid receptor antagonists	21 (10.5%)

Data are presented as mean ± standard deviation, median (interquartile range), or n (%)

**Fig. 3:**
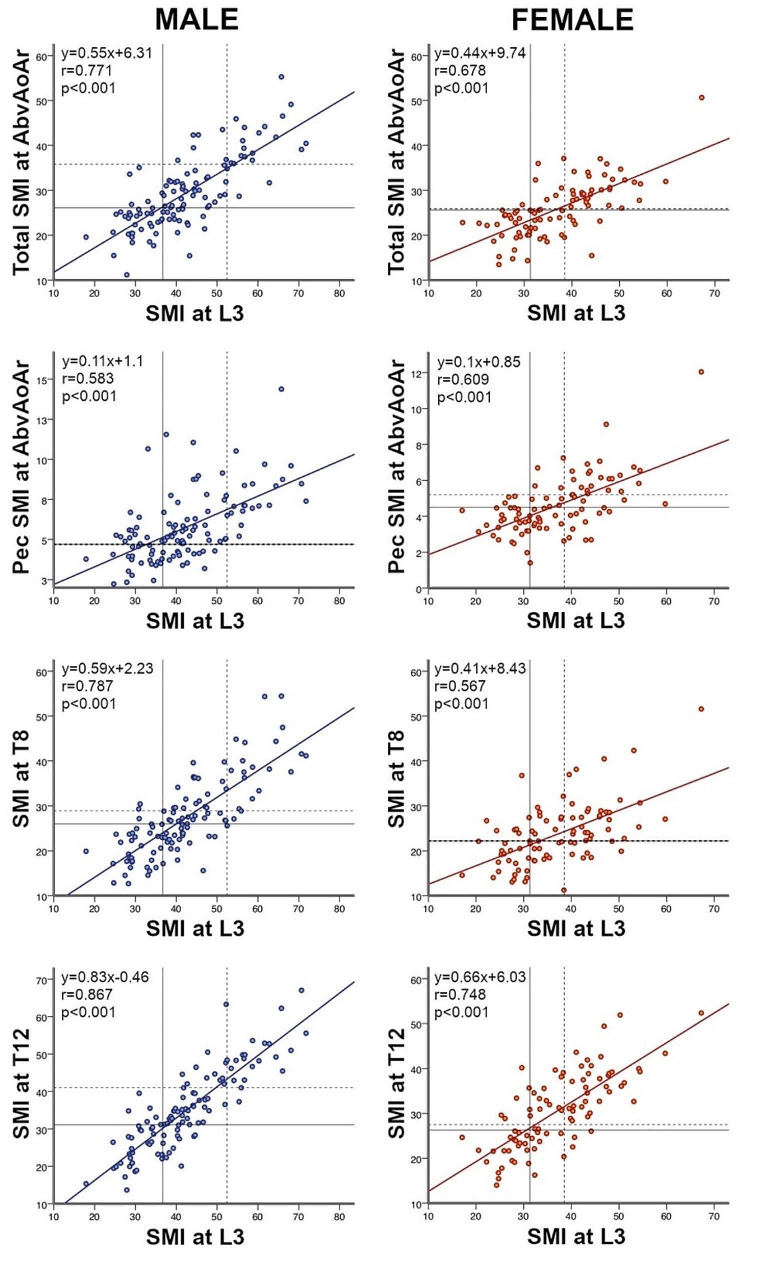
Scatterplots demonstrating the correlation between computed tomography L3 SMI measurements and those AbvAoAr (unilateral total and unilateral pectoralis), at T8, and at T12 in men (left) and women (right). Horizontal and vertical lines represent the lowest sex-stratified tertile L3 SMI cutoffs (solid lines) and frequently used cancer L3 SMI cutoffs (dotted lines). SMI, skeletal muscle index; AbvAoAr, above the aortic arch; L3, third lumbar vertebra; Pec, pectoralis; T8, eighth thoracic vertebra; T12, twelfth thoracic vertebra

The strongest correlation of thoracic SMI at the different thoracic levels with the widely used L3 SMI was at the T12 level (Table 2). This was followed, in descending order, by total unilateral AbvAoAr, T8, and pectoralis unilateral AbvAoAr. The T12 level persisted as the strongest correlation after sex-stratification (Fig. 3), but T8 was better than total unilateral AbvAoAr in men (*r* = 0.787 vs. *r* = 0.771), and pectoralis unilateral AbvAoAr was better than T8 in women (*r* = 0.609 vs. *r* = 0.567). A trend similar to SMI applied to IMAT%. The strongest correlation was at T12, followed by total unilateral AbvAoAr and T8; the correlation was weak at pectoralis unilateral AbvAoAr (Table 2).

**Table 2:** Correlations between computed tomography-derived skeletal muscle quantity and quality measurements at various thoracic vertebral levels compared to the third lumbar vertebral level (L3), with group comparisons based on sex-specific tertiles of L3 skeletal muscle index

Parameters	Correlation (*r*) with L3	*P*-value	Lowest L3 SMI tertile (*n* = 65)	Middle L3 SMI tertile (*n* = 67)	Highest L3 SMI tertile (*n* = 68)	*P*-value
**Skeletal muscle index (SMI) (cm**^**2**^/m^**2**^**)**						
Total unilateral AbvAoAr	0.754	**< 0.001**	22.3 ± 4.2	27.2 ± 5.2	33.7 ± 7.3	**< 0.001**
Pectoralis unilateral AbvAoAr	0.619	**< 0.001**	3.9 (3.4–4.7)	4.9 (4.0-5.8)	6.3 (5.0-7.4)	**< 0.001**
Total bilateral T8	0.726	**< 0.001**	20.2 ± 4.9	25.2 ± 5.4	31.5 ± 8.6	**< 0.001**
Total bilateral T12	0.834	**< 0.001**	24.7 ± 6.0	31.1 ± 5.7	42.6 ± 8.3	**< 0.001**
Total bilateral L3	-	-	28.9 ± 4.1	38.7 ± 3.6	51.6 ± 7.6	**< 0.001**
**Intermuscular adipose tissue percentage (IMAT%) (%)**						
Total unilateral AbvAoAr	0.676	**< 0.001**	19.8 ± 8.9	18.9 ± 7.1	17.8 ± 8.2	0.374
Pectoralis unilateral AbvAoAr	0.374	**< 0.001**	1.0 (0.3–3.2)	1.4 (0.4–3.6)	1.4 (0.4–3.4)	0.485
Total bilateral T8	0.666	**< 0.001**	25.7 ± 10.9	27.5 ± 9.0	28.7 ± 10.6	0.234
Total bilateral T12	0.757	**< 0.001**	20.7 ± 8.5	21.7 ± 7.6	19.6 ± 8.6	0.351
Total bilateral L3	-	-	19.1 ± 8.4	17.7 ± 6.8	13.0 ± 6.6	**< 0.001**

L3, third lumbar vertebra; SMI, skeletal muscle index; AbvAoAr, above the aortic arch; T8, eighth thoracic vertebra; T12, twelfth thoracic vertebra

Next, a subgroup analysis was performed to assess the difference in correlation of L3 measurements to total unilateral AbvAoAr in those with (*n* = 27) and without (*n* = 173) a CIED present. The correlation of SMI and IMAT%, respectively, were *r* = 0.833 (*p* < 0.001) and *r* = 0.809 (*p* < 0.001) in those with a CIED compared to *r* = 0.725 (*p* < 0.001) and *r* = 0.716 (*p* < 0.001) in those without a CIED. These correlations were compared using Fisher Z-transformation without a significant difference for SMI (z = 1.28, *p* = 0.200) or IMAT% (z = 1.03, *p* = 0.303).

Finally, patients were grouped based on sex-stratified tertile cutoffs of SMI at L3 into lowest, middle, and highest tertiles. Detailed data for the groups are presented in Table S2. A total of 65 patients (32.5%) were identified as being in the lowest tertile. These patients were older with lower weight, BSA, and BMI than the other groups. There was no difference in sex, race, or HF classification. N-terminal prohormone of brain natriuretic peptide (NT-proBNP) was higher, and albumin was lower among patients in the lowest tertile. The only difference in comorbidities and medications was higher statin use among patients in the highest and middle tertiles. The average SMI differed at all levels between the groups, but the average IMAT% only differed at L3 (Table 2). Area under the ROC curve analysis was used to obtain low thoracic vertebral SMI cutoffs based on the sample’s lowest sex-stratified tertile L3 SMI cutoffs (65/200) and the frequently used cancer L3 SMI cutoffs (142/200), which are presented in Table 3 along with their sensitivities and specificities.

**Table 3:** Thoracic computed tomography cutoffs for low skeletal muscle index based on sex-stratified tertile and frequently used cancer cutoffs at the third lumbar vertebra

	Female (*n* = 89)					Male (*n* = 111)				
Parameters	Cutoff	Sensitivity (%)	Specificity (%)	Area under the ROC	*P*-value	Cutoffs	Sensitivity (%)	Specificity (%)	Area under the ROC	*P*-value
**Cutoffs based on the lowest sex-stratified L3 SMI tertile (cm** ^2^ **/m** ^**2**^ **)**										
Total unilateral AbvAoAr	25.6	96.6	66.7	0.848	**< 0.001**	26.1	86.1	81.3	0.876	**< 0.001**
Pectoralis unilateral AbvAoAr	4.5	89.7	60.0	0.779	**< 0.001**	4.7	63.9	81.3	0.799	**< 0.001**
Total bilateral at T8	22.2	75.9	68.3	0.789	**< 0.001**	26.0	91.7	70.7	0.872	**< 0.001**
Total bilateral at T12	26.3	79.3	81.7	0.859	**< 0.001**	31.1	86.1	84.0	0.902	**< 0.001**
Total bilateral at L3^†^	31.3	-	-	-	-	36.7	-	-	-	-
**Cutoffs based on frequently used cancer L3 cutoffs (cm** ^**2**^ **/m** ^**2**^ **)**										
Total unilateral AbvAoAr	25.9	86.3	86.8	0.860	**< 0.001**	35.8	93.4	90.0	0.945	**< 0.001**
Pectoralis unilateral AbvAoAr	5.2	92.2	60.5	0.794	**< 0.001**	4.7	39.6	100.0	0.883	**< 0.001**
Total bilateral at T8	22.2	66.7	78.9	0.775	**< 0.001**	28.9	78.0	95.0	0.945	**< 0.001**
Total bilateral at T12	27.5	64.7	92.1	0.853	**< 0.001**	41.0	86.8	95.0	0.953	**< 0.001**
Total bilateral at L3^‡^	38.5	-	-	-	-	52.4	-	-	-	-

ROC, receiver operating characteristic; L3, third lumbar vertebra; SMI, skeletal muscle index; AbvAoAr, above the aortic arch; T8, eighth thoracic vertebra; T12, twelfth thoracic vertebra

^†^ Lowest sex-stratified tertile cutoffs for skeletal muscle index at the third lumbar vertebra derived from the analytic sample

^‡^ Frequently used cancer cutoffs for skeletal muscle index at the third lumbar vertebra from Prado et al. 2008

## Discussion

Our study comparing single-slice skeletal muscle evaluation at the widely used L3 level on abdominopelvic CT with immediately above the aortic arch, T8, and T12 on chest CT demonstrates that SMI (muscle quantity) at T12 has the strongest correlation with L3. This was also true for IMAT% (muscle quality); however, the average IMAT% among the L3 SMI tertiles was only significantly different at L3, making it potentially less valuable at the other levels. Thus, based on these results, T12 may be a good target for opportunistic muscle evaluation in patients with HF without CT of the abdomen/pelvis to predict outcomes or diagnose sarcopenia or malnutrition. The cutoffs based on the lowest sex-stratified L3 SMI tertile were 26.3 cm^2^/m^2^ for females and 31.1 cm^2^/m^2^ for males at the T12 level, while the cutoffs based on the frequently used cancer L3 SMI cutoffs were 27.5 cm^2^/m^2^ for females and 41.0 cm^2^/m^2^ for males at the T12 level.

Early sarcopenia identification is critical for intervention and mitigation of poor outcomes, but a formal diagnosis can be complex, given the need for muscle strength testing [11]. Opportunistic evaluation of skeletal muscles on imaging alone to identify myopenia is useful for outcomes prediction and early recognition of muscle wasting [19]. However, the widely used abdominopelvic muscle measurements at L3 [11] are of low opportunistic use in cardiology, where most imaging is done of the chest.

Unfortunately, there is significant heterogeneity in muscle measurements of the chest used in the literature, with most data derived from oncology and pulmonology populations [20]. A notable 2017 study compared SMI at L3 to T12 and T7 on preoperative CT of the aorta in patients undergoing transcatheter aortic valve replacement (TAVR) [21]. They found a higher correlation at T12 (*r* = 0.709, *p* < 0.001), which agrees with our study, indicating T12 as the most likely candidate for opportunistic use in cardiology patients. The close correlation of muscle area at the third or fourth lumbar vertebral levels with whole-body muscle measurements has been postulated to be due to the psoas, paraspinal, and abdominal muscles at these levels being minimally influenced by activity, unlike the appendicular muscles [22]. Thus, the close correlation seen at T12 may be due to its proximity to the lumbar vertebrae with the presence of abdominal muscles at the level; although T12 does not contain psoas, muscles seen at this landmark include rectus abdominis, diaphragm, external oblique, intercostals, latissimus dorse, and erector spinae.

A unique aspect of thoracic imaging in cardiology, particularly HF, is the presence of CIEDs, which may impede the assessment of adjacent structures due to metal artifacts in CT studies. Prior studies have addressed this limitation by making unilateral measurements opposite the device implantation site [23], a technique we also utilized in our study. Despite this, 26 patients were excluded from the original sample due to significant artifacts extending to both sides. To ensure that the presence of devices did not alter the correlation of upper thoracic measurements with L3, we performed a subgroup analysis of patients with versus without CIEDs. Both groups showed good correlation without a significant difference between them, indicating that the contralateral artifact, not extending beyond the midline subjectively, did not alter HUs, and thus measurements, in the upper chest.

Given the widespread use of L3 SMI, various cutoff values have been proposed. The first and most used cutoffs were established in 2008 in an obese Canadian population with respiratory or gastrointestinal cancers [17]. Sex-specific cutoffs of 52.4 cm²/m² in men and 38.5 cm²/m² in women were found based on association with mortality. Despite this, most studies derive their own cutoff values from morbidity and mortality or sex-specific lowest tertile, quartile, or fifth percentile of subjects, particularly outside of oncology [22]. Given the lack of cutoffs in cardiology populations, the latter definitions are likely more appropriate.

The previously mentioned 2017 study in patients undergoing TAVR utilized the frequently used L3 SMI cancer cutoff established in 2008 and proposed T12 cutoffs of 42.6 cm^2^/m^2^ in men and 30.6 cm^2^/m^2^ in women [21]. We focused on using the lowest sex-stratified L3 SMI tertile for low muscle mass evaluation with T12 SMI cutoffs of 31.1 cm²/m² in men and 26.3 cm²/m² in women. In addition, we also provided cutoffs at T12 of 41.0 cm²/m² in men and 27.5 cm²/m² in women based on those L3 SMI cancer cutoffs. The cancer cutoffs identified a much higher number of patients as having low muscle mass than sex-stratified tertiles (71.0% vs. 32.5%). However, an outcomes-based evaluation of these cutoffs in larger samples is required to compare their utility in cardiology patients.

Among the grouped participants based on sex-stratified L3 SMI tertiles, a significant difference was seen in NT-proBNP and albumin levels. The median NT-proBNP among patients in the lowest tertile was more than double that of the other groups. This agrees with prior studies showing a strong inverse relationship between SMA and NT-proBNP [24]. Although this difference could also be attributed to the higher BMI in patients without low muscle mass, multiple studies have demonstrated lean rather than fat mass to be responsible for the association between higher BMI and lower NT-proBNP [24–26]. Although the mechanism is unclear, the influence of sex steroid hormones has been postulated [27]. The association between low muscle mass and hypoalbuminemia is also not surprising, given the close relationship between myopenia and malnutrition, both contributing to frailty and worse outcomes [28].

The association between low muscle mass and lower statin use in our study is less clear. Statins have well-known benefits for primary and secondary prevention of cardiovascular disease, but they can induce adverse effects on the muscles. Although statin use post-endovascular aortic repair has shown lower long-term mortality without predisposition to muscle wasting based on successive SMA measurements [29], statin-induced myopathy shares some proposed mechanisms with myopenia and sarcopenia in HF, such as mitochondrial dysfunction [30] and ubiquitin-proteasome system upregulation [31, 32]. This leads us to believe that the lower statin use in patients with myopenia may be due to intolerance from worsened myopathy instead of statin use being protective against muscle wasting.

There are several limitations to our study. Although the study sample was more than most myopenia or sarcopenia studies, it was still small without power or sample size calculations, making type II error possible. Several patients also had to be excluded, primarily because of issues with opportunistic CT windows; this may be addressed by incorporating standardized institutional scanning protocols on routine imaging to ensure adequate windows containing all tissues. Measurements were done using the update to ABACS, ABACS+, for which there is limited validation, particularly on vertebral levels other than L3. Measurements AbvAoAr also had to be made unilaterally, given the presence of unilateral artifacts in most patients. The sample was obtained from patients hospitalized for ADHF, which may alter study variables; however, data such as weight and creatinine were obtained from the last values before discharge to ensure euvolemia and homeostasis. Finally, the diagnosis of sarcopenia was not made formally, given the lack of muscle strength testing.

In conclusion, frequently obtained imaging studies, such as computed tomography, are of opportunistic use for body composition analysis in chronic diseases like heart failure. However, such evaluation has been difficult in cardiac patients given the current consensus of using third or fourth lumbar vertebrae on abdominopelvic imaging while most imaging in cardiology is of the chest. This study adds to the limited literature supporting using the twelfth thoracic vertebra as a landmark for skeletal muscle evaluation. This allows us to further expand our knowledge on myopenia and sarcopenia in patients with heart failure and work towards a consensus for skeletal muscle evaluation in this population.

## Electronic supplementary material

Below is the link to the electronic supplementary material.

Supplementary Material 1

## Data Availability

The authors had full access to all the data in the study, take responsibility for the integrity of the data and accuracy of the data analysis, and may agree to make data available under special circumstances.
