# Supplementary material for: Skeletal muscle quantity and quality evaluation in heart failure: comparing thoracic versus abdominopelvic CT approaches
Source: Int J Cardiovasc Imaging. 2024 Jul 4;40(8):1787–96. doi: 10.1007/s10554-024-03169-w (PMC11401786; doi:10.1007/s10554-024-03169-w)
Supplement: Supplementary file 1 — Supplementary Material 1 [file 10554_2024_3169_MOESM1_ESM.docx]

**Supplemental Materials**

**Title**: Skeletal Muscle Quantity and Quality Evaluation in Heart Failure: Comparing Thoracic Versus Abdominopelvic CT Approaches

**Short Title**: Thoracic CT Muscle in Heart Failure

**Authors**: Saeid Mirzai, DO,^1,2^ Ian Persits, DO,^1^ Pieter Martens, MD, PhD,^3^ Jerry D. Estep, MD,^4^ W. H. Wilson Tang, MD,^3^ Po-Hao Chen, MD, MBA^5^

**Affiliations**:

^1^ Department of Internal Medicine, Cleveland Clinic, Cleveland, OH, USA

^2^ Section on Cardiovascular Medicine, Department of Internal Medicine, Wake Forest University School of Medicine, Winston-Salem, NC, USA

^3^ Kaufman Center for Heart Failure Treatment and Recovery, Heart Vascular and Thoracic Institute, Cleveland Clinic, Cleveland, OH, USA

^4^ Department of Cardiology, Cleveland Clinic Florida, Weston, FL, USA

^5^ Section of Musculoskeletal Imaging, Diagnostics Institute, Cleveland Clinic, Cleveland, OH, USA

**Corresponding Author**:

Po-Hao Chen, MD, MBA; Assistant Professor, Lerner College of Medicine at Case Western Reserve University; Section of Musculoskeletal Imaging, Diagnostics Institute, Cleveland Clinic, 9500 Euclid Avenue, Desk JJ36, Cleveland, OH 44195; Phone: (216) 445-6593 / E-mail: chenp2@ccf.org

**Supplemental Text**:

None.

**Supplemental Reference Section**:

**Table S1**. Intraobserver agreements for computed tomography body composition measurements.

**Table S2**. Comparison of baseline characteristics among groups separated based on sex-stratified tertile cutoffs of skeletal muscle index, normalized via height squared, at the third lumbar vertebra.

**Supplemental Tables**:

| **Table S1**. Intraobserver agreements for computed tomography body composition measurements. | |
| --- | --- |
| **Parameters** | **Intraclass correlation coefficient (95% confidence interval)**^†^ |
| **Skeletal muscle index (SMI) (cm^2^/m^2^)** | |
| Total unilateral AbvAoAr | 0.999 (0.997-1.000) |
| Pectoralis unilateral AbvAoAr | 0.982 (0.929-0.996) |
| Total bilateral T8 | 0.999 (0.995-1.000) |
| Total bilateral T12 | 0.992 (0.968-0.998) |
| Total bilateral L3 | 0.996 (0.986-0.999) |
| **Intermuscular adipose tissue percentage (IMAT%) (%)** | |
| Total unilateral AbvAoAr | 0.997 (0.988-0.999) |
| Pectoralis unilateral AbvAoAr | 0.702 (-0.198-0.926) |
| Total bilateral T8 | 0.996 (0.984-0.999) |
| Total bilateral T12 | 0.992 (0.966-0.998) |
| Total bilateral L3 | 0.973 (0.889-0.993) |
| **Footnote**: AbvAoAr, above the aortic arch; T8, eighth thoracic vertebra; T12, twelfth thoracic vertebra; L3, third lumbar vertebra.  ^†^ The intraobserver variability analysis was performed on raw skeletal muscle area and intermuscular adipose tissue measurements rather than the indices and percentages, respectively. | |

| **Table S2**. Comparison of baseline characteristics among groups separated based on sex-stratified tertile cutoffs of skeletal muscle index, normalized via height squared, at the third lumbar vertebra. | | | | |
| --- | --- | --- | --- | --- |
| **Parameters** | **Lowest L3 SMI tertile (n=65)** | **Middle L3 SMI tertile (n=67)** | **Highest L3 SMI tertile (n=68)** | **P-value** |
| **Characteristics** | | | | |
| Age (years) | 74±13 | 72±13 | 67±14 | **0.003** |
| Female sex | 29 (44.6%) | 30 (44.8%) | 30 (44.1%) | 0.997 |
| Race |  |  |  | 0.588 |
| Caucasian | 53 (81.5%) | 51 (76.1%) | 47 (69.1%) |  |
| Black | 10 (15.4%) | 13 (19.4%) | 17 (25.0%) |  |
| Other | 2 (3.1%) | 3 (4.5%) | 4 (5.9%) |  |
| Height (cm) | 170.4±10.4 | 168.0±10.0 | 169.2±12.3 | 0.440 |
| Weight (kg) | 70.4±16.7 | 79.3±17.8 | 94.1±22.6 | **<0.001** |
| BSA (m^2^) | 1.82±0.24 | 1.91±0.23 | 2.09±0.30 | **<0.001** |
| BMI (kg/m^2^) | 24.2±5.2 | 28.2±6.3 | 32.8±6.9 | **<0.001** |
| LV ejection fraction |  |  |  | 0.998 |
| Preserved | 33 (50.8%) | 32 (47.8%) | 33 (48.5%) |  |
| Mildly reduced | 7 (10.8%) | 8 (11.9%) | 8 (11.8%) |  |
| Reduced | 25 (38.5%) | 27 (40.3%) | 27 (39.7%) |  |
| NT-proBNP (pg/mL) | 6896 (3840-16338) | 4586 (1520-13414) | 3303 (989-7348) | **0.001** |
| Hemoglobin A1c (%) | 6.2±1.0 | 6.2±1.1 | 6.7±1.6 | 0.162 |
| LDL (mg/dL) | 71.8±32.0 | 79.6±38.4 | 72.2±33.1 | 0.670 |
| Albumin (g/dL) | 3.3±0.6 | 3.3±0.6 | 3.6±0.5 | **0.001** |
| **Comorbidities** | | | | |
| Hypertension | 52 (80.0%) | 57 (85.1%) | 64 (94.1%) | 0.054 |
| Hyperlipidemia | 42 (64.6%) | 52 (77.6%) | 53 (77.9%) | 0.142 |
| Diabetes | 25 (38.5%) | 38 (56.7%) | 37 (54.4%) | 0.074 |
| CABG/PCI | 15 (23.1%) | 18 (26.9%) | 10 (14.7%) | 0.212 |
| PAD | 24 (36.9%) | 23 (34.3%) | 19 (27.9%) | 0.524 |
| CKD | 22 (34.4%) | 28 (41.8%) | 35 (51.5%) | 0.137 |
| Atrial fibrillation | 35 (53.8%) | 34 (50.7%) | 26 (38.2%) | 0.159 |
| CRT | 3 (4.6%) | 3 (4.5%) | 1 (1.5%) | 0.533 |
| COPD | 29 (44.6%) | 25 (37.3%) | 29 (42.6%) | 0.677 |
| Cirrhosis | 3 (4.6%) | 8 (11.9%) | 5 (7.4%) | 0.292 |
| Cancer history | 28 (43.1%) | 24 (35.8%) | 29 (42.6%) | 0.632 |
| Smoking |  |  |  | 0.286 |
| Active | 13 (21.3%) | 11 (17.2%) | 15 (23.1%) |  |
| Prior | 34 (55.7%) | 27 (42.2%) | 31 (47.7%) |  |
| Never | 14 (23.0%) | 26 (40.6%) | 19 (29.2%) |  |
| **Medications** | | | | |
| Statin | 28 (43.1%) | 36 (53.7%) | 44 (64.7%) | **0.044** |
| ACEi/ARB | 25 (38.5%) | 30 (44.8%) | 31 (45.6%) | 0.664 |
| Beta-blocker | 39 (60.0%) | 38 (56.7%) | 41 (60.3%) | 0.897 |
| MRA | 5 (7.7%) | 5 (7.5%) | 11 (16.2) | 0.171 |
| **Footnote**: Data are presented as mean ± standard deviation, median (interquartile range), or n (%). Abbreviations: BSA, body surface area; BMI, body mass index; LV, left ventricular; ABSI, a body shape index; NTproBNP, N-terminal prohormone of brain natriuretic peptide; LDL, low-density lipoprotein; CABG, coronary artery bypass grafting; PCI, percutaneous coronary intervention; PAD, peripheral arterial disease; CKD, chronic kidney disease; CRT, cardiac resynchronization therapy; COPD, chronic obstructive pulmonary disease; ACEi, angiotensin-converting enzyme inhibitors; ARB, angiotensin receptor blockers; MRA, mineralocorticoid receptor antagonists. | | | | |

**Supplemental Figures**:

None.
